# Neuronal Plasticity and Multisensory Integration in Filial Imprinting

**DOI:** 10.1371/journal.pone.0017777

**Published:** 2011-03-10

**Authors:** Stephen Michael Town, Brian John McCabe

**Affiliations:** Sub-department of Animal Behaviour, Department of Zoology, University of Cambridge, Cambridge, United Kingdom; Université Pierre et Marie Curie, France

## Abstract

Many organisms sample their environment through multiple sensory systems and the integration of multisensory information enhances learning. However, the mechanisms underlying multisensory memory formation and their similarity to unisensory mechanisms remain unclear. Filial imprinting is one example in which experience is multisensory, and the mechanisms of unisensory neuronal plasticity are well established. We investigated the storage of audiovisual information through experience by comparing the activity of neurons in the intermediate and medial mesopallium of imprinted and naïve domestic chicks (*Gallus gallus domesticus*) in response to an audiovisual imprinting stimulus and novel object and their auditory and visual components. We find that imprinting enhanced the mean response magnitude of neurons to unisensory but not multisensory stimuli. Furthermore, imprinting enhanced responses to incongruent audiovisual stimuli comprised of mismatched auditory and visual components. Our results suggest that the effects of imprinting on the unisensory and multisensory responsiveness of IMM neurons differ and that IMM neurons may function to detect unexpected deviations from the audiovisual imprinting stimulus.

## Introduction

The integration of information from several sensory modalities offers an enriched perception of the world and provides a more robust method for representing and recognizing objects. Multisensory integration increases information content and disambiguates information that might otherwise have multiple interpretations [1]. Furthermore, integrating multisensory information enhances the reliability of sensory estimates [2], [3] and increases the speed of perceptual learning [4], [5].

How is multisensory information stored in the brain? The neuronal basis of multisensory integration has been investigated in several behaviors [6], [7], [8]. However, relatively few studies have directly assessed the effect of experience on the neuronal representation of multisensory information: Familiar and unfamiliar audiovisual stimuli evoke differential activation of the posterior superior temporal sulcus [9], left inferior frontal cortex, intraparietal sulcus [10], occipitotemporal junction and parahippocampal gyrus [11]. However, at the level of the single neuron, comparable studies of stimulus familiarity are lacking and therefore the neuronal basis of multisensory memory formation remains unclear.

Additionally, one would like to know whether the mechanisms underlying the storage of unisensory and multisensory information resemble one another. Despite the prevalence of multisensory stimuli in the natural world, many studies of object recognition have investigated the representation of unisensory information [12], [13], [14]. It therefore remains to be tested whether the findings of these studies extend to multisensory information and whether one can explain multisensory information storage in terms of the storage of information about its unisensory components.

To address these questions, we studied an animal model of object recognition; filial imprinting, in which young birds learn to recognize an audiovisual stimulus [15]. We recorded neurons from a critical forebrain region [16], [17]; the intermediate and medial mesopallium (IMM) of imprinted and naïve domestic chicks during presentation of an audiovisual imprinting stimulus and novel object, and of their auditory and visual components. We presented a fully balanced stimulus set (Table 1) that included incongruent audiovisual combinations, in which the auditory and visual components of an imprinting stimulus and novel object were mismatched. This experimental design allowed us to compare the effect of imprinting on unisensory and multisensory neuronal responses and to investigate the nature of any multisensory representation formed through experience. We find that imprinting enhanced the mean magnitude of neuronal response to unisensory components of the imprinting stimuli but not to the multisensory imprinting stimulus itself. Rather imprinting most strongly enhanced the response of neurons to a mismatched audiovisual stimulus combining the visual component of the imprinting stimulus and auditory component of a novel object.

**Table 1:** Stimulus set presented to each animal with abbreviations.

	Visual Component			
Auditory component	None (Unisensory)	Imprinting stimulus	Novel Object	
None (Unisensory)	Not Applicable	**V_IS_**	**V_NO_**	
Imprinting stimulus	**A_IS_**	**A_IS_V_IS_**	**A_IS_V_NO_**	
Novel Object	**A_NO_**	**A_NO_V_IS_**	**A_NO_V_NO_**	

Each stimulus was presented for 4 seconds 15–20 times with a minimum inter-stimulus interval of 4 s.

## Results

We recorded activity from 157 neurons in the IMM of three imprinted and three naive chicks (see Methods) during presentation of an audiovisual imprinting stimulus (IS) and novel object (NO) and their auditory and visual components. We characterized the response of each neuron to each stimulus using response magnitude - the firing rate during presentation expressed as a percentage of the baseline firing rate measured before presentation.

### Visual Stimuli (V_IS_ and V_NO_)

**Figure 1:**
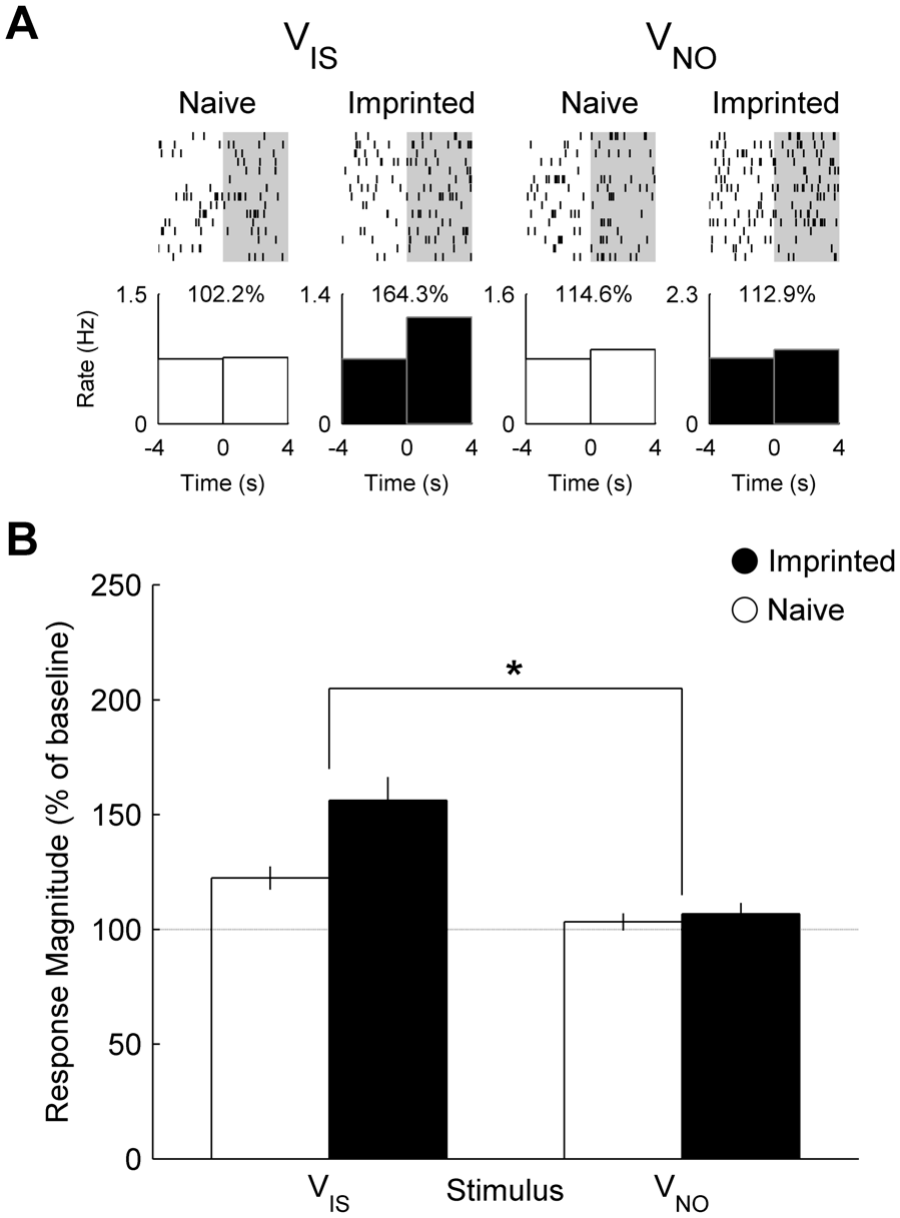
Single cell and population responses to visual stimuli. (**A**) Raster plot and peri-stimulus time histograms illustrating the firing rate of two neurons recorded from a naïve and an imprinted chick before and during presentation of the visual components of the imprinting stimulus (V_IS_) and novel object (V_NO_). Percentage values indicate the response magnitude calculated as the firing rate during presentation (0 to 4 s) expressed as a percentage of pre-stimulus baseline firing rate (−4 to 0 s). (**B**) Mean (± s.e.m.) response magnitude of neuronal populations recorded in naïve (white: n = 85) and imprinted (black: n = 72) chicks. (*) indicates a significant interaction between group and stimulus (*P* = 0.015).

We found that neurons recorded from imprinted chicks responded more strongly to the visual component of the imprinting stimulus (V_IS_) than the visual component of the novel object (V_NO_) whereas neurons recorded from naïve chicks did not. **Figure 1a** shows the firing rate of two neurons recorded from an imprinted chick and a naïve chick before and during presentation of visual stimuli. Across the neuronal population (**Fig. 1b**), we found that the mean response magnitude was significantly greater towards V_IS_ than V_NO_ in the neurons recorded from imprinted by not naïve chicks (ANOVA, interaction between effects of group and stimulus: *F*_1, 155_ = 6.01, *P* = 0.015).

### Auditory Stimuli (A_IS_ and A_NO_)

**Figure 2 pone-0017777-g002:**
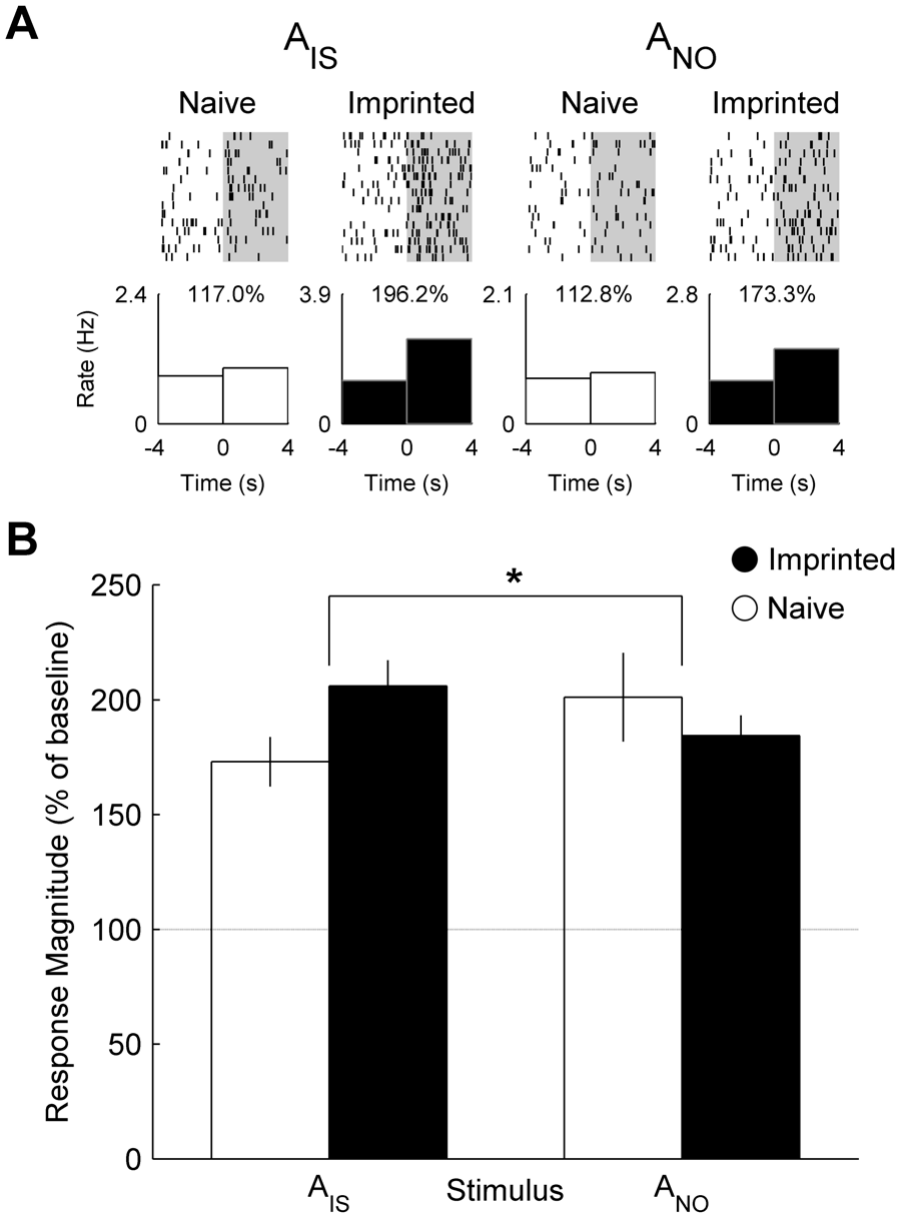
Single cell and population responses to auditory stimuli. (**A**) Raster plot and peri-stimulus time histograms illustrating the firing rate of the same neurons shown in figure 1 before and during presentation of the auditory components of the imprinting stimulus (A_IS_) and novel object (A_NO_). Percentage values indicate the response magnitude calculated as the firing rate during presentation (0 to 4 s) expressed as a percentage of pre-stimulus baseline firing rate (−4 to 0 s). (**B**) Mean (± s.e.m.) response magnitude of neuronal populations recorded in naïve (white) and imprinted (black) chicks. (*) indicates a significant interaction between group and stimulus (*P* = 0.017).

Similarly, the neuron presented in **figure 1a** recorded from an imprinted chick responded more strongly to the auditory component of the imprinting stimulus (A_IS_) than that of the novel object (A_NO_) whereas the neuron recorded from a naïve chick showed little difference in response between stimuli (**Fig. 2a**). Across the recorded populations, the mean response of neurons from imprinted chicks to A_IS_ was greater than to A_NO_ whereas the mean response of neurons from naïve chicks to A_IS_ was weaker than to A_NO_ (**Fig. 2b**) (interaction between group and stimulus: *F*_1, 155_ = 5.86, *P* = 0.017).

### Congruent Audiovisual Stimuli (A_IS_V_IS_ and A_NO_V_NO_)

**Figure 3:**
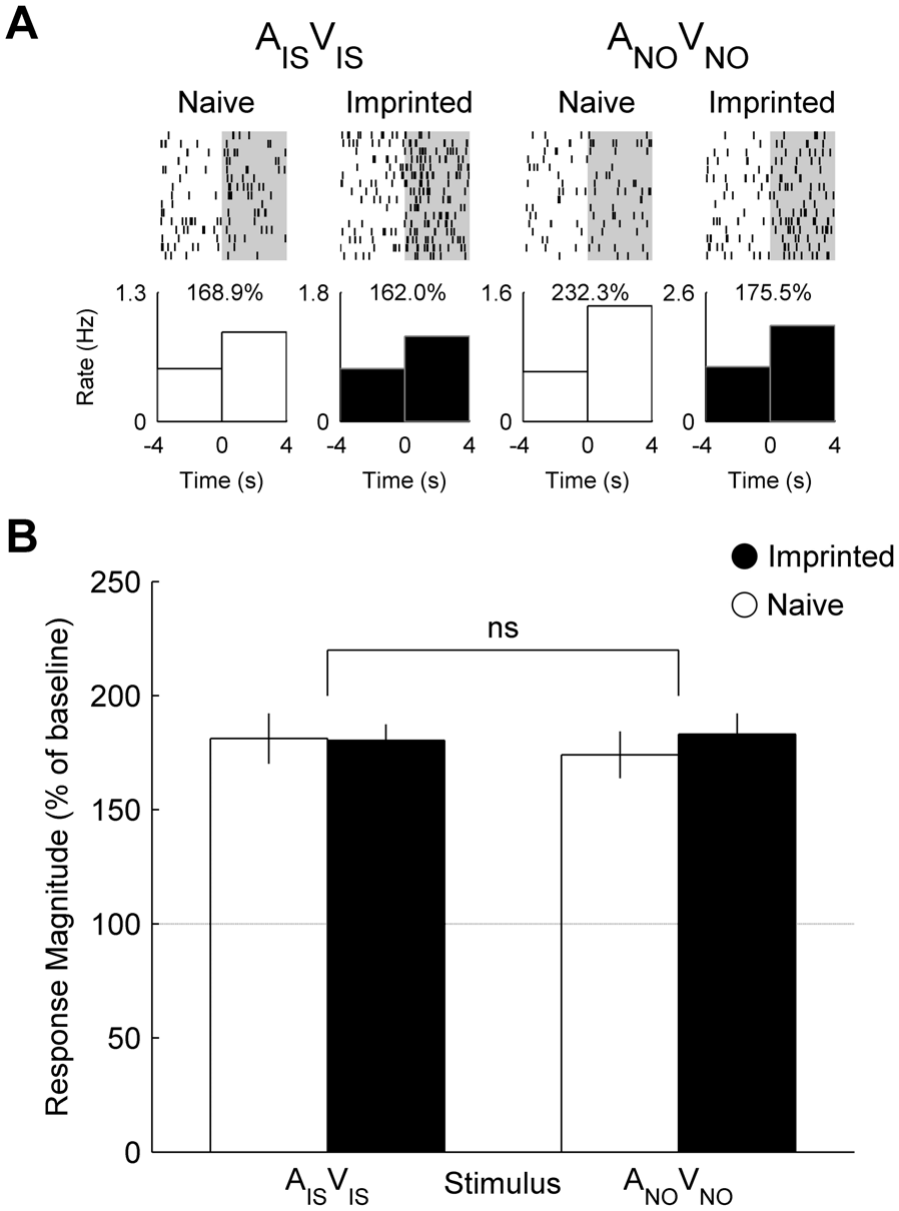
Single cell and population responses to congruent audiovisual stimuli. (**A**) Raster plot and peri-stimulus time histograms illustrating the firing rate of the same neurons from figures 1 and 2 before and during presentation of the audiovisual imprinting stimulus (A_IS_ V_IS_) and novel object (A_NO_ V_NO_). Percentage values indicate the response magnitude calculated as the firing rate during presentation (0 to 4 s) expressed as a percentage of pre-stimulus baseline firing rate (−4 to 0 s). (**B**) Mean (± s.e.m.) response magnitude of neuronal populations recorded in naïve (white) and imprinted (black) chicks. (ns) indicates the absence of interaction between group and stimulus.

In contrast, single neurons recorded from the imprinted chick such as that presented in **Fig. 1** and **Fig. 2** responded similarly to the audiovisual imprinting stimulus (A_IS_V_IS_) and novel object (A_NO_V_NO_) and there was little difference in response to the audiovisual imprinting stimulus between neurons recorded from imprinted and naïve chicks (**Fig. 3a**). Comparison between neuronal populations recorded from imprinted and naïve chicks revealed no main effect of group or stimulus and no interaction between these factors (**Fig. 3b**).

**Figure 4:**
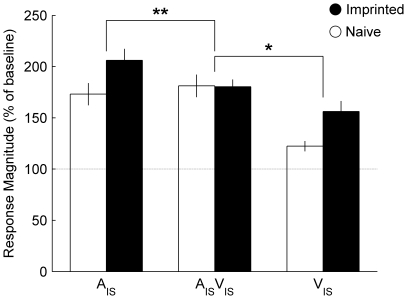
Comparison between the effects of imprinting on different modalities. Mean (± s.e.m.) response magnitude to the audiovisual imprinting stimulus (A_IS_V_IS_) and its auditory (A_IS_) and visual components (V_IS_). (**) indicates interaction between the effects of imprinting on auditory and audiovisual stimuli (*P* = 0.006). (*) indicates interaction between the effects of imprinting on auditory and audiovisual stimuli (*P* = 0.049).

The effect of imprinting on mean response magnitude to the imprinting stimulus varied with modality (**Fig. 4**): Comparison between visual and audiovisual modalities demonstrated that enhancement of mean response magnitude to V_IS_ was absent for A_IS_V_IS_ (interaction between group and modality; *F*_1, 155_ = 3.93, *P* = 0.049). Similarly, comparison between auditory (A_IS_) and audiovisual (A_IS_V_IS_) modalities demonstrated that the enhancement of mean response magnitude was limited to auditory stimuli (*F*_1, 154_ = 7.91, *P* = 0.006). Thus imprinting leads to the modification of neuronal responses that are limited to the unisensory components of the imprinting stimulus. Comparison between the effects of imprinting on responses to the audiovisual novel object and its auditory or visual components revealed no interactions between modality and group.

### Incongruent Audiovisual Stimuli (A_IS_V_NO_ and A_NO_V_IS_)

**Figure 5:**
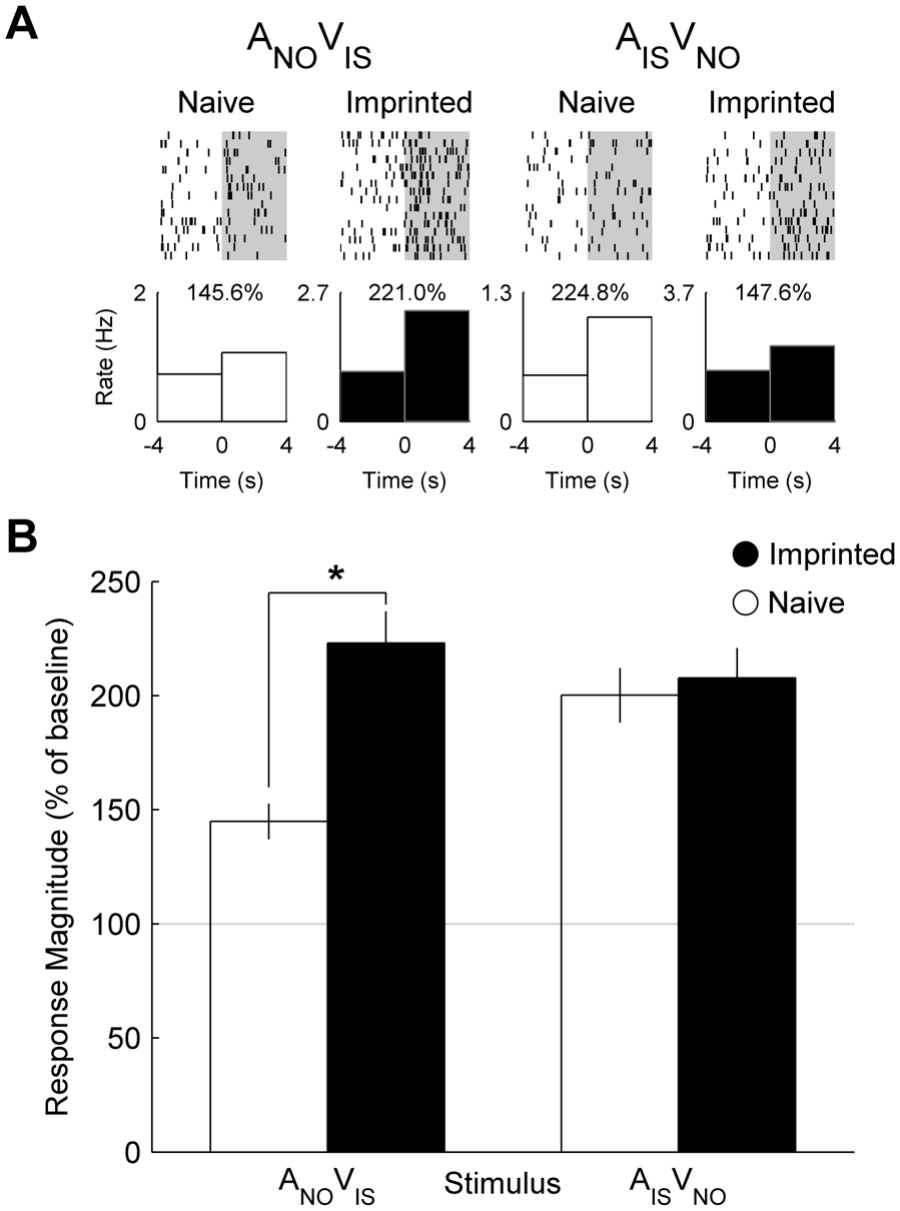
Single cell and population responses to incongruent audiovisual stimuli. (**A**) Raster plot and peri-stimulus time histograms illustrating the firing rate of the same neurons from figures 1–3 before and during presentation of incongruent audiovisual stimulus (A_NO_ V_IS_ and A_IS_ V_NO_). Percentage values indicate the response magnitude calculated as the firing rate during presentation (0 to 4 s) expressed as a percentage of pre-stimulus baseline firing rate (−4 to 0 s). (**B**) Mean (± s.e.m.) response magnitude of neuronal populations recorded in naïve (white) and imprinted (black) chicks. (*) indicates a significant effect of group on mean response magnitude to A_NO_ V_IS_ (*P*<0.001).

By mismatching the auditory and visual components of the imprinting stimulus and novel object, it was possible to create two incongruent audiovisual stimuli (A_IS_V_NO_ and A_NO_V_IS_). We found that single neurons recorded from imprinted chicks responded more strongly to the combination A_NO_V_IS_ than neurons recorded from naïve chicks (**Fig. 5a**). Comparing neuronal populations revealed a significant effect of group (*F*_1, 155_  = 26.23, *P*<0.001) (**Fig. 5b**). However, there was no effect of imprinting on the mean response to the alternative incongruent combination A_IS_V_NO_ (*P*>0.1).

### Multisensory Integration

**Figure 6:**
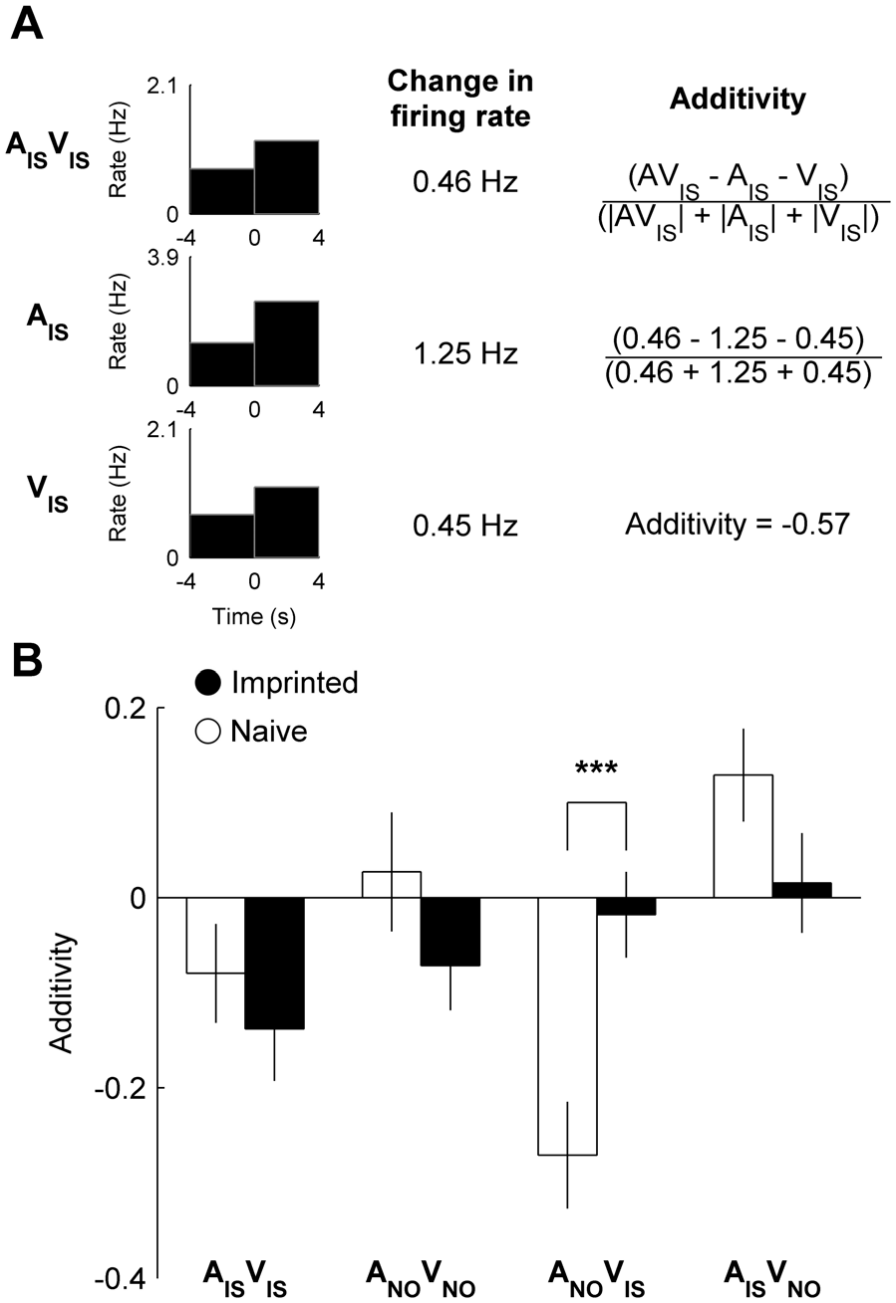
Experience-dependent audiovisual integration. (**A**) The response of a neuron recorded from an imprinted chick to the audiovisual imprinting stimulus (A_IS_V_IS_) and its auditory (A_IS_) and visual components (V_IS_) (see also Figs. 1–3). The change in firing rate was calculated for each stimulus and used to calculate the additivity index. (**B**) Mean (± s.e.m.) additvity index of neuronal populations recorded from naive and imprinted chicks to the audiovisual imprinting stimulus (A_IS_V_IS_) and novel object (A_NO_V_NO_) and to incongruent audiovisual stimuli (A_NO_V_IS_ and A_IS_V_NO_). (***) indicates significant effect of group on additivity index of A_NO_V_IS_ (*P*<0.001).

We also investigated whether multisensory integration was affected by imprinting by calculating an additivity index for each neuron for each audiovisual stimulus. In order to calculate additivity, we measured the change in firing rate during presentation of an audiovisual stimulus (AV) and its auditory (A) and visual (V) components (**Fig. 6a**). We then calculated the difference between the change in firing rate during presentation of AV and the sum of changes in firing rate during presentation of A and V, and divided this by the total sum of changes in firing rate (see **Fig. 6a** and Methods). The resulting variable therefore ranges from −1 to 1 with values greater than and less than zero indicate subadditivity and superadditivity respectively, whereas zero indicates that the change in firing rate during presentation of the audiovisual stimulus is equal to the sum of changes in firing rated during presentation of its auditory and visual components.

**Figure 6b** shows the mean additivity index for neurons recorded from naïve and imprinted chicks for each audiovisual stimulus. In accordance with our earlier findings, imprinting led to the increase in additivity in the case of incongruent responses to A_NO_V_IS_ (*T*_153_ = 4.68, *P*<0.001). This result can be explained by the strong increase in audiovisual response induced by imprinting, coupled with the weaker increase in familiar visual response and decrease in unfamiliar auditory response. The difference in mean additivity between groups was not significant for any of the other audiovisual stimuli.

## Discussion

### Unisensory and multisensory neuroplasticity

We report an imprinting-related enhancement in responses of neurons within the IMM for the auditory and visual components of an imprinting stimulus but not the audiovisual imprinting stimulus itself. This leads us to conclude that imprinting-related enhancement of the response magnitude of IMM neurons is limited to the unisensory components of an imprinting stimulus and does not extend to the audiovisual compound.

Our findings are consistent with earlier reports of the selective enhancement of neuronal responsiveness to auditory and visual components of an imprinting stimulus [13], [18], [19]. Our findings also support preliminary results of more recent work in which the proportion of IMM neurons found to be responsive to the visual component of an imprinting stimulus increased following imprinting whereas the proportion responsive to the audiovisual imprinting stimulus did not (Nicol & Horn, *Proceedings of the Physiological Society* 2009 Cardiff, UK. Available at www.physoc.org/Proceedings: Last Accessed Jan 2011).

However, our findings conflict with an earlier study by Brown and Horn [18] in which the proportion of sites within the IMM responsive to an audiovisual imprinting stimulus increased with imprinting (albeit, this increase was smaller than that reported for the visual component of the imprinting stimulus). This finding led to the assumption that imprinting similarly affects responsiveness to the audiovisual imprinting stimulus and its visual component [20]. Our findings challenge this assumption and suggest that unisensory and multisensory stimuli cannot be considered equivalent in the study of the neurophysiological basis of filial imprinting. The disparity between the present findings and those of Brown and Horn cannot be attributed to the difference in measurement used to characterize neurons (proportion of responsive sites vs. response magnitude) because reanalysis of our data according to the same method confirmed our finding: imprinting enhanced the proportion of sites responsive to the visual component of the imprinting stimulus but not the audiovisual stimulus itself (Table 2). Furthermore, recent work by other investigators has also found a dissociation in the effects of imprinting on proportion of responsive neurons to the audiovisual imprinting stimulus and its visual component (Nicol & Horn, *Proceedings of the Physiological Society* 2009 Cardiff, UK. Available at www.physoc.org/Proceedings: Last Accessed Jan 2011). Our results may differ from those of Brown and Horn because of differences in recording method: In the current study, we used tetrodes to identify the responses of single neurons whereas the earlier results were obtained using multi-unit recordings of the activity of clusters of neurons. It is possible that multi-unit recordings are limited in their sensitivity as a particularly responsive neuron can cause an entire cluster to be identified as responsive when the majority of units are unresponsive. Tetrodes allow the separation of neurons within such a cluster and therefore may avoid such biases, providing a more sensitive index of neuronal activity that could explain the difference between past studies and the present findings.

**Table 2:** Proportion of neurons responsive to the audiovisual imprinting stimulus and its visual component.

Modality	Group	Proportion responsive	
Visual	Naïve	17/85	20.0%
	Imprinted	24/72	33.3%
Audiovisual	Naïve	39/85	45.9%
	Imprinted	33/72	45.8%

Responsive neurons were defined as those whose firing rate during stimulus presentation significantly differed from the baseline firing rate before stimulus presentation (T-test: see ref. [18] for details).

**Figure 7:**
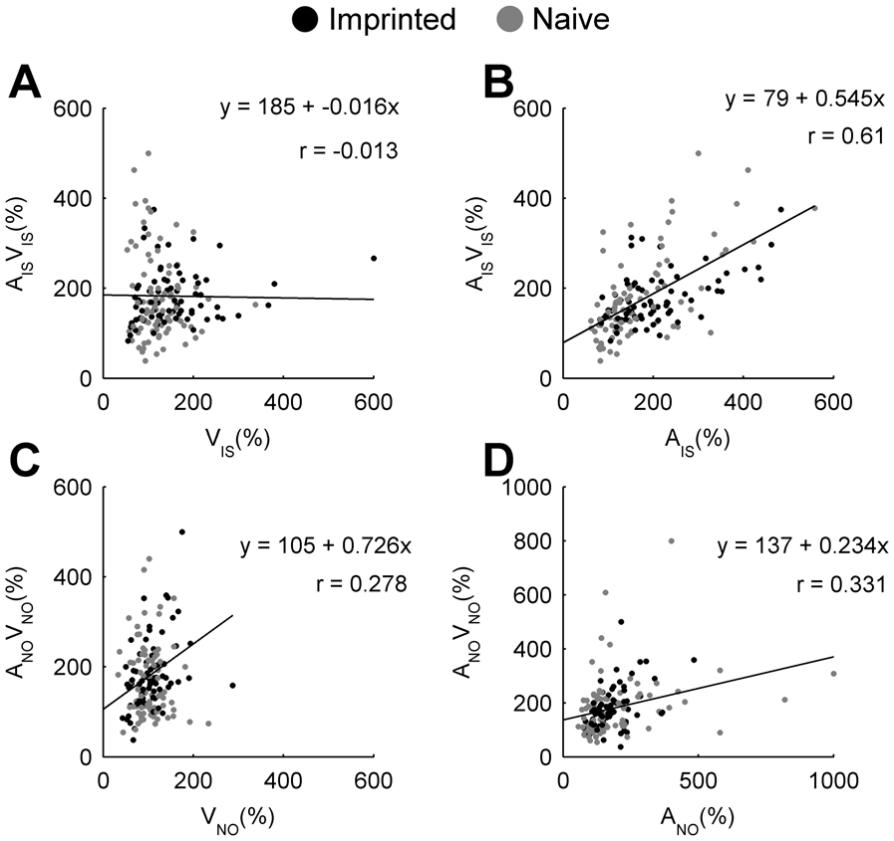
Principle of inverse effectiveness. Axes indicate the response magnitude (% of baseline activity) of individual neurons recorded from naïve (grey) and imprinted (black) chicks to unisensory (x-axis) and multisensory stimuli (y-axis). Equations and r-values indicate regression and correlation coefficients respectively. (**A**) Visual component (V_IS_) vs. audiovisual imprinting stimulus (A_IS_V_IS_). (**B**) Auditory component (A_IS_) vs. audiovisual imprinting stimulus (A_IS_V_IS_). (**C**) Visual component (V_NO_) vs. audiovisual novel object (A_NO_V_NO_). (**D**) Auditory component (A_NO_) vs. audiovisual novel object (A_NO_V_NO_).

The dissociation between changes in mean response magnitude to the multisensory imprinting stimulus and its unisensory components may be explained by the principle of inverse effectiveness. This principle describes the phenomenon occurring in both mammals and birds in which the effect of adding an additional modality to a stimulus on response magnitude is inversely proportional to the original salience of the stimulus when presented alone [22], [23], [24]. In the current study, the enhancement of neuronal responses to the auditory or visual components of the imprinting stimulus may lead to a reduction in the effectiveness of adding a second modality when the audiovisual imprinting stimulus is presented. This would lead to a relatively constant mean response magnitude to the audiovisual imprinting stimulus despite an increase in response magnitude to auditory and visual components, as we report. This interpretation is supported by the relatively weak correlation between response magnitude of neurons to the visual and audiovisual imprinting stimulus (**Fig. 7a**) and not novel object (**Fig. 7c**). However, there are strong correlations between the response of neurons to audiovisual stimuli and their auditory components, both for the imprinting stimulus (**Fig. 7b**) and the novel object (**Fig. 7d**). These correlations would not be predicted by the principle of inverse effectiveness; however it is possible that correlations are present because auditory stimuli are sufficiently salient that little enhancement through multisensory integration occurs anyway (**Fig. 4**). Under such circumstances, correlations between auditory and audiovisual responses might be expected as the audiovisual stimulus is no more salient than its auditory component.

**Figure 8:**
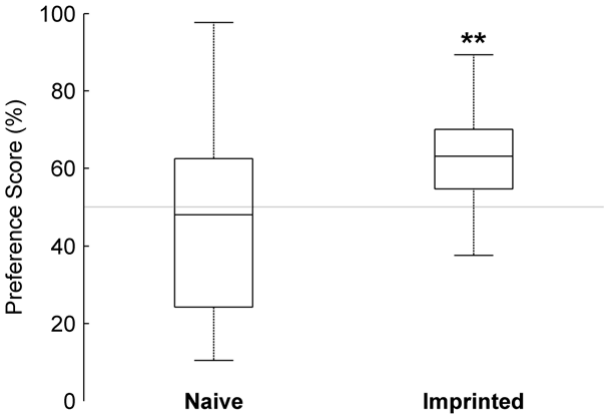
Behaviour during presentation of audiovisual stimuli. Preference scores for audiovisual imprinting stimulus. Box plots indicate median preference scores (center bar), upper and lower quartiles (box) and whiskers represent the range. (**) Comparison of individual medians revealed that the preferences for the audiovisual imprinting stimulus of imprinted (n = 8) but not naïve chicks (n = 9) were greater than chance (50%, sign test: naïve chicks, *P*>0.5; imprinted, *P* = 0.008).

The relationship between the behavior of chicks and the responses of IMM neurons to the audiovisual imprinting stimulus and novel object remains unclear. Imprinted but not naïve chicks were able to discriminate between the audiovisual imprinting stimulus and novel object (**Fig. 8**) yet the mean response magnitude of neurons to both stimuli in both groups were similar (**Fig. 4**). It therefore seems unlikely that response magnitudes of IMM neurons directly contribute to the discrimination between imprinting stimulus and novel object; rather our results suggest the neurons within the IMM may serve to identify unexpected auditory properties of the imprinting stimulus (see below).

### Audiovisual Incongruence

We report that the mean response magnitude to the incongruent audiovisual stimulus A_NO_V_IS_ (the visual component of the imprinting stimulus combined with the auditory component of the novel object) was greater in imprinted than naïve chicks. This result should be interpreted with caution as the difference in response magnitude derived mainly from the unusually weak responses to A_NO_V_IS_ recorded from neurons in naïve chicks. There is no prior reason to expect this audiovisual stimulus to differ so notably in its salience to naïve chicks from other audiovisual stimuli, raising the possibility that the finding is anomalous. However, there is also no reason to believe that the recording of neuronal responses to A_NO_V_IS_ was any less accurate than all other audiovisual stimuli: Presentation order was randomized across neuronal tests, making it unlikely that a consistent time of presentation biased the results. Furthermore, the analysis of neuronal activity and calculation of response magnitude following single unit isolation was automated for all stimuli, and therefore any inaccuracy in measurement of response magnitude for A_NO_V_IS_ should also be manifest in the measurement of response magnitude for all other stimuli. Moreover, it is also notable that of all audiovisual stimuli, A_NO_V_IS_ evoked the strongest mean response magnitude in the population recorded from imprinted chicks. We therefore believe that it is unlikely that the imprinting-related enhancement of mean response magnitude to A_NO_V_IS_ was anomalous but rather interpret the effect tentatively as a result of imprinting that may reveal important details regarding the function of IMM neurons; namely the detection of incongruous auditory accompaniments to the visual imprinting stimulus.

The suggestion that imprinting enhances neuronal responses to incongruous auditory accompaniments of the visual imprinting stimulus is consistent with the finding that the mean response magnitude to the visual component of the imprinting stimulus (V_IS_) was stronger in imprinted than naïve birds, as presentation of V_IS_ in the absence of any call could be considered an incongruent auditory condition given the original imprinting exposure was to the audiovisual compound A_IS_V_IS_. The suggestion is also consistent with the more general proposal that neurons in the IMM respond to unexpected variations from the original imprinting experience as neurons in the IMM also respond more strongly to A_IS_V_IS_ when presented in an unfamiliar than familiar visual context following imprinting (Town & McCabe, Unpublished). It remains to be seen whether this hypothesis accurately predicts the effects of imprinting on neuronal responses to more ethologically relevant stimuli such as live hens and naturalistic situations.

### Multisensory integration in the IMM

IMM neurons recorded from imprinted chicks responded strongly to auditory stimuli and to the visual component of the imprinting stimulus demonstrating that, at least following imprinting, information from multiple sensory modalities is integrated in the IMM. The ability of IMM neurons to respond to multiple modalities of sensory information is consistent with previous findings [18], [19] and the projection of afferents from visual (optic tectum, arcopallium intermedium, nidopallium and the Wulst) and auditory (Field L) regions of the brain to the IMM [25]. The afferents sent to the IMM from the nidopallium may also convey somatosensory information [25] and chicks show the ability to imprint on tactile information [26]. It would therefore be interesting to test whether IMM neurons also respond to somatosensory stimuli and whether these responses are dependent upon imprinting.

When calculating additivity in the IMM, we found that mean values of neurons recorded from imprinted chicks were near zero for the incongruent audiovisual stimulus A_NO_V_IS_ (mean ± s.e.m  =  −1.78±4.5; comparison vs. 0: *P*>0.5). This suggests that on average, the sum of changes in neuronal activity during presentation of the audiovisual stimulus was similar to the sum of changes in neuronal activity during separate presentation of its unisensory components. Mean additivity was also near zero for the incongruent stimulus A_IS_V_NO_ and audiovisual imprinting stimulus; however neither is likely to reflect audiovisual integration at the population level because the visual component (V_NO_) of the stimulus did not evoke strong responses from neurons and therefore a mean additivity value near zero may reflect similar responses to audiovisual and auditory stimuli. Thus, at least during presentation of A_NO_V_IS,_ neurons may integrate visual and auditory information.

### Remaining Questions

At present it remains unclear how incongruence detection is performed, or what its function might be. Additionally, it is unclear how auditory and visual information may be integrated, at least during the response of neurons recorded from imprinted chicks to A_NO_V_IS_. Much of this obscurity stems from the lack of structural and biochemical knowledge about neurophysiologically characterized neurons.

In terms of incongruence detection and multisensory integration, the underlying mechanisms will depend upon the form in which information reaches the IMM. It is not clear whether auditory and visual information are provided through separate (auditory and visual) or mixed (auditory, visual and audiovisual) channels. Recent evidence has demonstrated that the optic tectum, a structure thought to provide visual input to the IMM, is capable of multisensory integration in the Barn Owl [23]. Therefore, it may be likely that IMM neurons receive unisensory and multisensory information at synapses from structures that were originally described as unimodal. This speculation remains to be confirmed and will require paired recordings of neurons from the IMM and their presynaptic inputs from other regions of the brain. Furthermore, understanding the computations performed during synaptic integration within the dendritic tree will require the application of techniques such as *in-vivo* calcium-imaging to measure post-synaptic potentials at multiple synapses in deep tissue of behaving animals.

With regard to the function of incongruence detection, future studies will need to elucidate the regions of the brain to which specific neurons, responding most strongly to A_NO_V_IS_ and V_IS_, project. Neurons may project axons locally within the IMM or to relatively distant cognitive and motor regions such as the hyperpallium apicale, arcopallium (homologous to the mammalian amygdala) and striatum [25] and therefore imprinting-related responses of an IMM neuron to A_NO_V_IS_ and V_IS_ may serve one or more of several functions (e.g. social recognition, emotional behavior or generation of motor output) depending on its innervations pattern. It is notable therefore, that imprinted chicks did not differ greatly in the extent to which they approached A_NO_V_IS_ or A_IS_V_IS_ (i.e. congruent and incongruent stimuli) during stimulus presentations suggesting that the mean response magnitude of neurons within the IMM and approach behavior in the training wheel are not directly linked (median approach distance: A_IS_V_IS_  =  1.25 m, A_NO_V_IS_  =  1.23 m). The nature of synapses in innervated regions will also be of crucial importance in understanding the function of IMM neurons; within the IMM there is an imprinting-related enhancement of potassium stimulated GABA (γ-aminobutyric acid) release suggesting that imprinting may alter the balance of inhibition within the IMM [27]; however understanding how changes in inhibitory synapses affect the circuits in which IMM neurons take part will require knowledge of the neurophysiological properties of pre- and post-synaptic neurons.

### Conclusions

In summary, we report a dissociation between the effects of imprinting on the responses of IMM neurons to an audiovisual imprinting stimulus and its auditory and visual components, challenging the existing assumption that the effects of imprinting on unisensory and multisensory responsiveness are equivalent. We report an enhancement in mean response magnitude to an incongruent audiovisual stimulus, suggesting that neurons within the IMM may signal incongruous auditory accompaniments to the visual component of the imprinting stimulus. In future, it will be important to simultaneously characterize the neurophysiological, structural and biochemical properties of neurons in order to better understand the function of the IMM during imprinting.

## Materials and Methods

### Subjects

Subjects were domestic chicks (*Gallus gallus domesticus*, Ros 308 Strain) incubated and reared in darkness within incubators maintained at 32–35°C. All procedures were performed in accordance with the UK Animals (Scientific Procedures) Act 1986, under the UK Home Office Project License No. 80/2276 and were approved by the University Biomedical Support Services (UBSS) ethical review committee, University of Cambridge.

### Imprinting

Approximately 24 hours after hatching, 30 chicks were imprinted using methods similar to those described elsewhere [16]. Briefly; chicks were placed in running wheels within a darkened training box maintained at 30°C and exposed to an imprinting stimulus - a rotating, illuminated red box presented in conjunction with a maternal hen call (Call A, see below for further details about stimuli) - for two sessions of 60 minutes separated by an hour interval in which chicks were returned to incubators. Imprinted chicks were then identified by their ability to discriminate between the imprinting stimulus and novel object (a rotating, illuminated blue cylinder) in a sequential preference test in which chicks were returned to training wheels and presented with the visual component of the imprinting stimulus and novel object for two periods of four minutes in an ABBA design in which stimulus order was counterbalanced; neither stimulus was accompanied by explicit auditory stimulation. For each presentation, the distance the chick ran was measured and a preference score calculated as the distance run towards the imprinting stimulus (IS) as a percentage of the total distance run during the test (IS +NO):

Preference  =  100 × [IS / (IS + NO)]

For the imprinted group, only chicks with strong preferences for the imprinting stimulus (>70%: n = 8) were selected for surgical implantation of microelectrodes. Chicks in the naïve group (n = 9) remained in a holding incubator and received no exposure to the imprinting stimulus prior to implantation.

### Microelectrode Design and Implantation

Neuronal activity was recorded using four platinum/iridium wires (Neuralynx, Bozeman, MT, USA) wound together and bonded (tetrodes)[21]. In order to penetrate the brain, tetrodes were mounted onto thin (dia. 125 µm) tungsten wire (Advent Research Materials, Oxford, UK) with cyanoacrylate superglue. The resultant structure was then fixed into a guide cannula and the protruding end coated in 1,1′-dioctadecyl−3,3,3′,3′-tetramethylindocarbocyanine perchlorate (DiI) (Sigma); a neuronal tracer allowing electrode localization [28]. The tetrode tips were then gold-plated to an impedance of 0.2–0.4 MΩ prior to surgery.

Chicks were anaesthetized (0.12 ml Equithesin, intraperitoneal)[29] and positioned in a sterotaxic frame. A craniotomy was performed 0.8 mm lateral to the midline and 2.5 mm anterior to the frontoparietal suture and the dura mater removed. A microdrive assembly [30] was then glued to the dorsal surface of the skull, allowing one tetrode to be positioned over the left or right IMM. A reference electrode was also placed under the skull permitting differential recording and the assembly was stabilized in dental cement. At the end of surgery, each tetrode was then advanced approximately 1.25 mm over a period of 2.5 hrs.

### Neuronal recording

Following recovery from surgery overnight, neuronal activity was detected in the awake animal, placed in a modified running wheel in which a tether connected the microdrive to recording equipment: Signals were amplified 10,000 times, band-pass filtered between 300 and 3,000 Hz (CyberAmp; Axon Instruments, Union City, CA, USA) and sampled at 14 kHz for offline analysis (Power1401 laboratory interface and Spike2; Cambridge Electronic Design, Cambridge, UK). Tetrodes were advanced until spontaneous neuronal activity was detected and chicks were then presented with familiar and unfamiliar visual, auditory and audiovisual stimuli.

Detailed accounts of visual and auditory stimuli can be found elsewhere [16], [31]. Briefly; the visual stimulus was either a red and black box (9×17.5×18 cm; l×w×h), or a blue and white cylinder (diameter, 15.5 cm; height, 19 cm). Both were illuminated from within by 24 W bulbs, rotated at 30 revolutions per minute and placed 65 cm from the running wheel. During stimulus presentation, current was provided to the stimuli to cause illumination and rotation. Between presentations, the stimuli were dim and static and elicited little interest from the animals. Auditory stimuli were maternal calls (Calls A and B) recorded from two hens and presented at approximately 75 dB using a cassette player controlled by a TTL pulse from the Power1401 laboratory interface and a pair of loud speakers placed out of view of the animal. Audiovisual stimuli consisted of all possible combinations of visual and auditory components of the imprinting stimulus and novel object (Table 1).

All stimulus presentations lasted four seconds and throughout presentation of visual and audiovisual stimuli, chicks were required to look towards the visual stimulus with both eyes during presentation. This was ensured by monitoring head position via video camera and excluding presentations in which either or both eyes were turned away from the stimulus. Stimuli were presented in a consecutive sequence an average of 15 times and the stimulus order was randomized between chicks. The approach behavior of the chick was also recorded during stimulus presentation and this data was used to confirm the ability of subjects to discriminate between audiovisual as well as visual stimuli: For each four second presentation, the distance run during presentation was measured and preference scores calculated using the mean distance run towards the audiovisual imprinting stimulus and novel object.

Following a testing session, tetrodes were advanced at least 200 µm to avoid repeated sampling of the same neurons. Chicks were then returned to holding incubators for at least 45 minutes between tests. In six birds, spontaneous activity was not satisfactorily detected at any depth and therefore only behavioral data from these birds were analyzed.

### Electrode Localization

At the end of the experiment, chicks were euthanized (0.1 ml Euthatal, intraperitoneal) and perfused transcardially with 0.9% saline and 4% paraformaldehyde in PBS (Sigma). The brain was removed and stored in 4% paraformaldehyde in PBS until 24 hours before sectioning, at which point the brain was transferred to 20% sucrose (Sigma). Frozen sections were then cut at 180 µm thickness and tetrode location confirmed by the presence of DiI stained neurons. Data from five subjects were excluded because tetrodes were positioned outside the IMM.

### Data Analysis

Single units were isolated from recorded data using standard cluster cutting techniques [21], [32]. Briefly; events with amplitudes between two and ten times the background noise on at least one channel of the tetrode were selected by threshold detection (Spike2). Events were then sorted by waveform parameters and principal components using k-means and manual clustering. Events that did not resemble action potentials on at least one channel were discarded. Single unit isolation was assessed using spike interval histograms and visual inspection of waveform shape; the minimum interval between spikes was greater than 2 ms for all neurons. Following isolation, the times of stimulus presentation and spikes were saved and subsequently analyzed in Matlab (Mathworks, Natick, MA, USA).

Single-unit responses were then assessed using the normalized response magnitude, calculated as:

RM  =  100 × (P/B).

Where P is the mean firing rate of a neuron during the 4 s stimulus presentation, and B is the firing rate in the 4 s baseline period before presentation. Mean response magnitudes to the audiovisual imprinting stimulus and novel object, their auditory and visual components and incongruent audiovisual stimuli were compared between imprinted and naive birds in a 2×2 (stimulus × group) analysis of variance (ANOVA; Genstat, VSN International, Hemel Hempstead, UK). Modality replaced stimulus as a factor for comparisons between visual or auditory and audiovisual imprinting stimuli. By comparing naïve and imprinted birds it was possible to control for stimulus salience. By comparing neuronal responses to the imprinting stimulus and novel object or their unisensory components, it was possible to determine whether the effect of imprinting was generalized or specific to the imprinting stimuli previously experienced. Therefore by observing the interaction of group and stimulus, we could exclude the influences of stimulus salience or generalization from our interpretation. Regression and correlation coefficients used to describe the relationship between the magnitudes of responses to audiovisual stimuli and their auditory and visual components were calculated in Matlab.

For each neuron we also characterized the integration of auditory and visual information using the additivity index of multisensory integration [modified from 33]. In our index, additivity was calculated in two stages, firstly we calculated the corrected the firing rate of a neuron in response to an audiovisual stimulus (AV) by deducting the baseline firing rate before presentation (B_AV_) from the firing rate during presentation (P_AV_). The same corrections were also applied to responses to auditory (A) and visual (V) components of the audiovisual stimulus:

AV  =  P_AV_ − B_AV_

A  =  P_A_ − B_A_

V  =  P_V_ − B_V_

Corrected firing rates were then used in the following equation to calculate additivity:

Additivity  =  (AV − A − V) / (|AV| + |A| + |V|)

Denominator values were made absolute because the combination of positive and negative values (i.e. responses at a rate lower than baseline firing rate) could lead to cancellation that made the total sum of neuronal activity inaccurately low. For each audiovisual stimulus, the mean additivity index was compared between imprinted and naïve birds by t-test.
