# Correction: Neuronal Plasticity and Multisensory Integration in Filial Imprinting

**DOI:** 10.1371/annotation/23463d25-b95f-43cd-8e86-7db3c7ba977c

**Published:** 2011-08-23

**Authors:** Stephen Michael Town, Brian John McCabe

The authors wish to amend the incorrect reference to a paper in the Discussion section. The authors state: "We were incorrect to state (Discussion, third paragraph) that Brown and Horn [18] showed that the proportion of sites within the IMM responsive to an audiovisual imprinting stimulus increased with imprinting. Their paper makes no assertion about the effects of imprinting on neuronal responsiveness to an audiovisual imprinting stimulus. That paper could not, therefore, have "led to the assumption that imprinting similarly affects responsiveness to the audiovisual imprinting stimulus and its visual component." We were incorrect to make this assertion and to refer to it in the Conclusions. In light of these errors, for which we apologize, the third paragraph of the Discussion and the relevant part of the Conclusions should be disregarded." 

